# How soccer team students shape adolescent athlete engagement: evidence from an actor–partner interdependence model

**DOI:** 10.3389/fpubh.2026.1904710

**Published:** 2026-08-20

**Authors:** Haopeng Wang, Huarui Huang, Chen Zhong, Yizhou Shui

**Affiliations:** 1 School of Physical Education, Shaanxi Normal University, Xi’an, China; 2 School of Psychology, Shaanxi Normal University, Xi’an, China

**Keywords:** actor-partner interdependence model, adolescent, athlete engagement, athlete participation, soccer, social interaction

## Abstract

**Background:**

Physical inactivity among adolescents has become a global public health concern. Insufficient engagement in sports potentially affects physical and mental health. Athlete engagement, reflecting persistent positive emotional experiences in physical activity, is a key factor in promoting adolescent development. This study investigates how adolescent athlete participation influences athlete engagement within and across different student groups.

**Methods:**

A total of 4,140 students from 167 soccer-featured schools in China are recruited, comprising 2,070 soccer team students and 2,070 non-soccer team students. Participants complete the Athlete Participation Scale (APS) and the Athlete Engagement Questionnaire (AEQ). Data are analyzed using descriptive statistics, correlation analysis, and the Actor–Partner Interdependence Model (APIM) with latent variables to examine actor and partner effects.

**Results:**

Soccer team students exhibit a significant actor effect on their own engagement (*β* = 0.824, *p* < 0.001). However, they do not have a significant partner effect (*β* = −0.005, *p* > 0.05). In contrast, non-soccer team students exhibit a statistically significant actor effect. However, the effect size is small (*β* = 0.045, *p* < 0.05). Meanwhile, the partner effect is significant (*β* = 0.518, *p* < 0.001). The partner effect is stronger than the actor effect. The dyadic pattern analysis classifies the pattern for soccer team students as an actor-only pattern and the pattern for non-soccer team students as a mixed actor–partner pattern.

**Conclusion:**

Athlete participation influences adolescent athlete engagement differently across student groups. Soccer team students’ athlete engagement is mainly driven by their own athlete participation. For non-soccer team students, their own athlete participation has only a limited effect on their athlete engagement. Non-soccer team students are more strongly influenced by the role-modeling of soccer team students. These findings highlight the value of strengthening campus soccer teams to mobilize non-soccer team students’ sustained engagement in physical activity.

## 1 Introduction

Insufficient participation in sports among adolescents is an important global public health issue (1). The World Health Organization (WHO) reports that 80% of adolescents fail to achieve the recommended levels of sports (2). This issue is particularly salient in China. During adolescence, young people face many challenges, including growing academic and psychological pressures (3). These challenges make it more difficult for adolescents to participate in sports (4). Compared with individual sports, team sports provide greater social support and more opportunities for communication and collaboration. Therefore, team sports may be more beneficial to adolescent development (5). Among various team sports, soccer relies mainly on lower-limb movements to execute technical actions; these movements occur in body regions far from the brain. They may better activate cerebellar–prefrontal neural circuits linked to executive function. This may provide unique advantages for adolescent neural and brain development (6). It can be seen that soccer may play an important role in promoting sport and improving the quality of exercise among adolescents. Therefore, improving the quality of youth soccer participation is an important issue. The indicators used to assess participation quality also require further discussion.

In early research, sports are described using indicators such as intensity, duration, and frequency. However, research indicates that these indicators cannot fully reflect the benefits of sports on adolescent development (7). As research has progressed, athlete participation and athlete engagement have also become important indicators for assessing the level and quality of sports. They make a greater contribution to adolescent development (7). Athlete participation refers to an individual’s participation in a specific sports context. It includes three dimensions: behavioral participation, emotional participation, and cognitive participation (8). It reflects an individual’s level of participation in sports. Athlete engagement refers to a persistent, positive emotional experience in sports (9, 10). Its characteristics include confidence, dedication, vigor, and enthusiasm. It reflects the quality of sports. Research demonstrates that athlete participation positively predicts athlete engagement (11). However, a gap remains because existing research focuses on a specific group of professional adolescent athletes. Existing research does not explore the relationship between athlete participation and athlete engagement across different adolescent groups.

In Chinese campus soccer, soccer team students and non-soccer team students constitute the two main groups involved in athlete participation (12, 13). There is a clear distinction between these two groups. Soccer team students receive long-term professional soccer training and represent their schools in competitions (14). Non-soccer team students generally participate only in regular physical education classes. They complete course assignments and tests. Soccer team students and non-soccer team students have different identities and goals in athlete participation. Therefore, athlete participation may have different effects on athlete engagement in these two groups.

According to social interaction theory, individual behavior results from interaction among individuals (15). This theory also suggests that individuals are more likely to adopt a behavior when they place greater value on it (16). For soccer team students, communication with teammates fosters team belonging and self-identity. These factors further improve athlete engagement. For non-soccer team students, peer interaction increases their interest in sports and produces other positive outcomes. These benefits further promote their sport engagement.

Specifically, athlete participation among soccer team students occurs in a highly organized team environment; this environment may provide more opportunities for them to receive support and recognition from others (17, 18). Soccer requires students to cooperate with their teammates, which links individual behavior to collective performance (19). Teamwork can strengthen their responsibility, thereby improving their athlete engagement (20). In addition, mutual trust among teammates and a shared commitment to team goals can enhance athlete engagement (21, 22). On the other hand, non-soccer team students’ athlete participation may predict their athlete engagement through the positive values of sports. Non-soccer team students are less likely to participate in high-intensity team sports (23). However, school sports activities can also provide them with positive values, such as enjoyment and confidence (24, 25). These values can reduce the difficulties and pressures students face when participating in sports. Based on these, non-soccer team students’ athlete participation may also positively predict their athlete engagement.

In addition, social interaction theory suggests that individual social behavior is not an isolated phenomenon and is often influenced by other groups (26–28). In schools, adolescents’ participation in sport is often influenced by their peers. Soccer team students may receive positive feedback from their friends. This positive feedback can further motivate them to become more involved in soccer. Non-soccer team students may view soccer team members as role models. They may adjust their level of athlete engagement by observing soccer team students’ behavior.

Specifically, soccer team students represent their schools in competitions. They receive encouragement and support from their peers during these competitions (29). This may strengthen the social recognition of soccer team students, thereby reinforcing their athlete engagement (30). On the other hand, soccer team students serve as role models for non-soccer team students (31). The positive image of soccer team students may help non-soccer team students recognize the value of sports (32, 33). This may lead them to behave more like soccer team students, which may further increase their emotional and behavioral engagement in sport (34).

**Figure 1:**
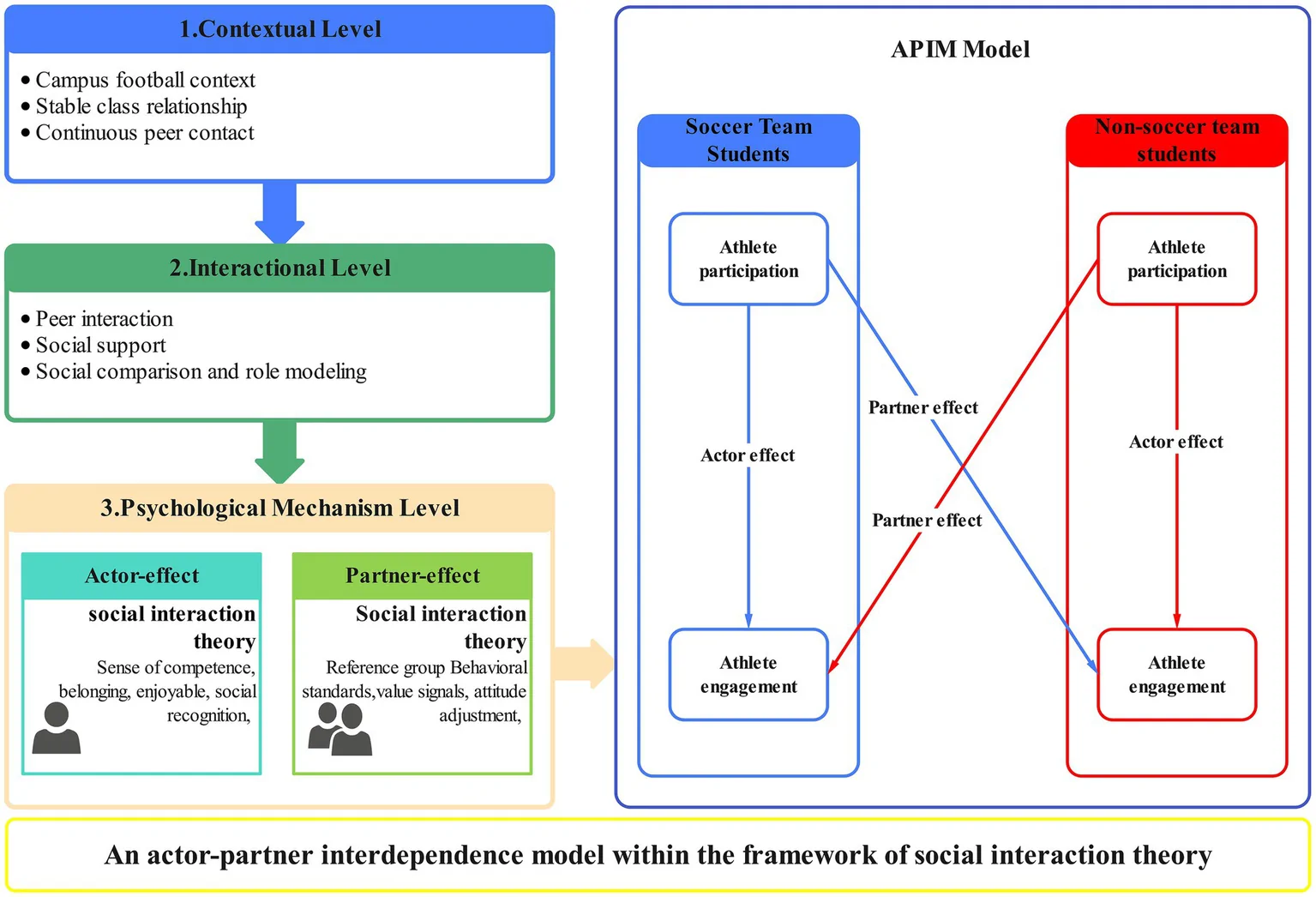
Theoretical framework.

Currently, no research examines the relationship between athlete participation and athlete engagement across different adolescent groups or the mutual influence among these groups. Based on social interaction theory, this study attempts to construct an actor-partner interdependence model for the first time. This study matches soccer team students with non-soccer team students on a one-to-one basis. The non-soccer team students have access to stable, long-term sports support. This study aims to use the unique advantages of soccer to promote healthy adolescent development. It also aims to improve the level and quality of adolescents’ athlete engagement. The following hypotheses are proposed. *H1a*: Athlete participation of soccer team students may positively predict their own athlete engagement (Actor effect). H1b: Soccer team students’ athlete engagement may be positively influenced by the athlete participation of non-soccer team students (Partner effect). *H2a*: Non-soccer team students’ athlete participation may positively predict their own athlete engagement (Actor effect). *H2b*: Non-soccer team students’ athlete engagement may be positively influenced by the soccer team students’ athlete participation (Partner effect) (see Figure 1).

## 2 Materials and methods

### 2.1 Participants

After written informed consent is obtained from all participants and their legal guardians, 2,143 primary and secondary school soccer team students are selected through the screening procedure. These students participate in after-school soccer training and competitions. Meanwhile, 2,143 non-soccer team students are selected. These students participate only in regular physical education classes and do not belong to any school sports team. The pairing is based on stable, naturally occurring friendships in the school sports context. The formation of each dyad is confirmed by both members. In addition, each non-soccer team student independently selects one soccer team student from the same class as their dyadic partner. They have the same gender. Meanwhile, each non-soccer team student receives long-term help in sports from the selected soccer team student. Long-term help refers to soccer team students providing non-soccer team students with one-on-one technical guidance during weekly soccer classes for at least one semester. They also maintain a close friendship that develops naturally and lasts for more than 2 years. A total of 4,286 questionnaires are collected. 73 dyads involving 146 questionnaires are excluded. The exclusion rate is 3.4%. Questionnaires are excluded for three reasons. First, soccer team students do not provide long-term help to non-soccer team students. Second, the two students do not maintain a stable friendship. Third, questionnaires involving one-to-many pairings are also excluded. A total of 2,070 dyadic datasets are obtained. The final sample consists of 4,140 students from 167 soccer-featured schools across China. The valid response rate is 96.6%. Detailed social demographic characteristics are presented in Table 1.

**Table 1:** Social demographic characteristics (*n* = 4,140).

Variable	Frequency	Percentage
Gender		
Male soccer team students	1,590	76.8%
Male non-soccer team students	1,590	
Female soccer team students	480	23.2%
Female non-soccer team students	480	
Level of school		
Primary school	2,312	55.8%
Junior high school	1,040	25.1%
Senior high school	788	19.1%

### 2.2 Measurement

#### 2.2.1 Basic information

The basic information questionnaire collects demographic data, including gender (1 = male, 2 = female), school level (1 = primary school, 2 = junior high school, 3 = senior high school), and school team membership (1 = yes, 2 = no). Additionally, this questionnaire is designed with skip questions: A soccer team student provides long-term assistance to a peer (Name: ____). A non-soccer team student receives long-term assistance from a soccer team student (Name: ____). A previous study shows that among demographic variables, only gender and school level can influence athlete engagement (7). Therefore, in this study, gender (1 = male, 2 = female) and school level (1 = primary school, 2 = junior high school, 3 = senior high school) are incorporated as covariates.

#### 2.2.2 Athlete participation scale (APS)

Athlete participation is assessed using the APS developed by Agbuga et al. (8). Zhang provides the Chinese translation of the APS (35). This scale consists of three dimensions: behavioral participation, emotional participation and cognitive participation. It includes 13 items. It uses a 5-point Likert scale, from 1 (strongly disagree) to 5 (strongly agree). In this study, Cronbach’s *α* is 0.941 for the APS, 0.834 for behavioral participation, 0.833 for emotional participation, and 0.844 for cognitive participation. Specifically, Cronbach’s α is 0.952 for soccer team students and 0.986 for non-soccer team students. Confirmatory factor analysis also indicates favorable model fit: χ^2^/df = 8.246, CFI = 0.980, TLI = 0.974, GFI = 0.963, RMSEA = 0.059.

#### 2.2.3 Athlete engagement questionnaire (AEQ)

Athlete engagement is measured using the AEQ (9, 10). The AEQ shows good reliability and validity in Chinese adolescents (36). This questionnaire consists of four dimensions: confidence, vitality, dedication and enthusiasm. It includes 16 items. It uses a 5-point Likert scale, from 1 (never) to 5 (always). In this study, Cronbach’s *α* is 0.954 for the AEQ, 0.856 for confidence, 0.840 for vitality, 0.840 for dedication, and 0.857 for enthusiasm. Specifically, Cronbach’s *α* is 0.943 for soccer team students and 0.952 for non-soccer team students. Confirmatory factor analysis also indicates favorable model fit: *χ*^2^/df = 7.721, CFI = 0.971, TLI = 0.965, GFI = 0.955, RMSEA = 0.057.

### 2.3 Data analysis

Descriptive statistics and paired-samples t-tests are performed using SPSS 28.0. Pearson correlation analysis is performed to examine the relationships between variables. The actor-partner interdependence model (APIM) is analyzed using Amos 26.0. Previous research shows that latent variable models can reduce measurement error and improve the accuracy of path estimation (37). Therefore, this study uses a latent-variable-based actor–partner interdependence model for analysis. Model fit is evaluated based on the comparative fit index (CFI), Tucker-Lewis index (TLI), standardized root mean square residual (SRMR), and root mean square error of approximation (RMSEA) (38). Concurrently, the confidence interval for the k-value of the actor–partner interdependence model (APIM) is estimated via 5,000 bootstrap resamples using Amos 26.0 to examine the dyadic patterns of the APIM (39).

## 3 Results

### 3.1 Common method bias test

This study uses the unmeasured latent method construct (ULMC) approach to assess common method variance (40). The original confirmatory factor analysis (CFA) model is compared with a CFA model incorporating a common method factor (CMF) that loads on all observed variables. The model fit of these two models is compared. The results reveal the following: ΔCFI = 0.006, ΔTLI = 0.004, ΔRMSEA = 0.004, SRMR = 0.029. The differences in CFI and TLI are less than 0.02, while the differences in RMSEA and SRMR are less than 0.05 (24, 41). Meanwhile, Harman’s single-factor test is used in this study to examine the percentage of variance explained by the common method factor. The results indicate that the percentage of variance explained by the common method factor is 33.956%. This value is below the critical threshold of 40%. Therefore, no significant common method bias is detected in this study, and the common method bias is unlikely to be a serious concern.

### 3.2 Descriptive statistics and paired-samples *t*-test

The descriptive statistics and paired-samples t-test are shown in Table 2. The results indicate that a significant difference exists between soccer team students and non-soccer team students in both athlete participation (*p* < 0.001) and athlete engagement (*p* < 0.001).

**Table 2:** Descriptive statistics and paired-samples *t*-test (*n* = 4,140).

Variable	Soccer (*M* ± SD)	Non (*M* ± SD)	Bootstrap 95% CI		*t*
Athlete Participation	57.83 ± 8.43	48.02 ± 17.59	8.97	10.65	22.9^***^
Athlete Engagement	69.75 ± 8.90	64.29 ± 11.39	5.29	5.64	62.13^***^

***p < 0.001, soccer means soccer team students; non means non-soccer team students.

### 3.3 Correlation analysis

Correlation analysis results are presented in Table 3. Athlete participation among soccer team students is significantly positively correlated with their own athlete engagement (*p* < 0.001) and the athlete engagement of non-soccer team students (*p* < 0.001). Athlete engagement among soccer team students is significantly positively correlated with athlete engagement among non-soccer team students (*p* < 0.001).

**Table 3:** Correlation analysis (*n* = 4,140).

Student groups	Variable	*M*	SD	1	2	3	4
Soccer team students	1. Athlete participation	57.826	8.427	1			
	2. Athlete engagement	69.751	8.904	0.892^***^	1		
Non-soccer team students	3. Athlete participation	48.017	17.594	0.002	−0.002	1	
	4. Athlete engagement	64.286	11.393	0.464^***^	0.489^***^	0.044^*^	1

*p < 0.05; ***p < 0.001.

### 3.4 Actor-partner interdependence model analysis

**Figure 2:**
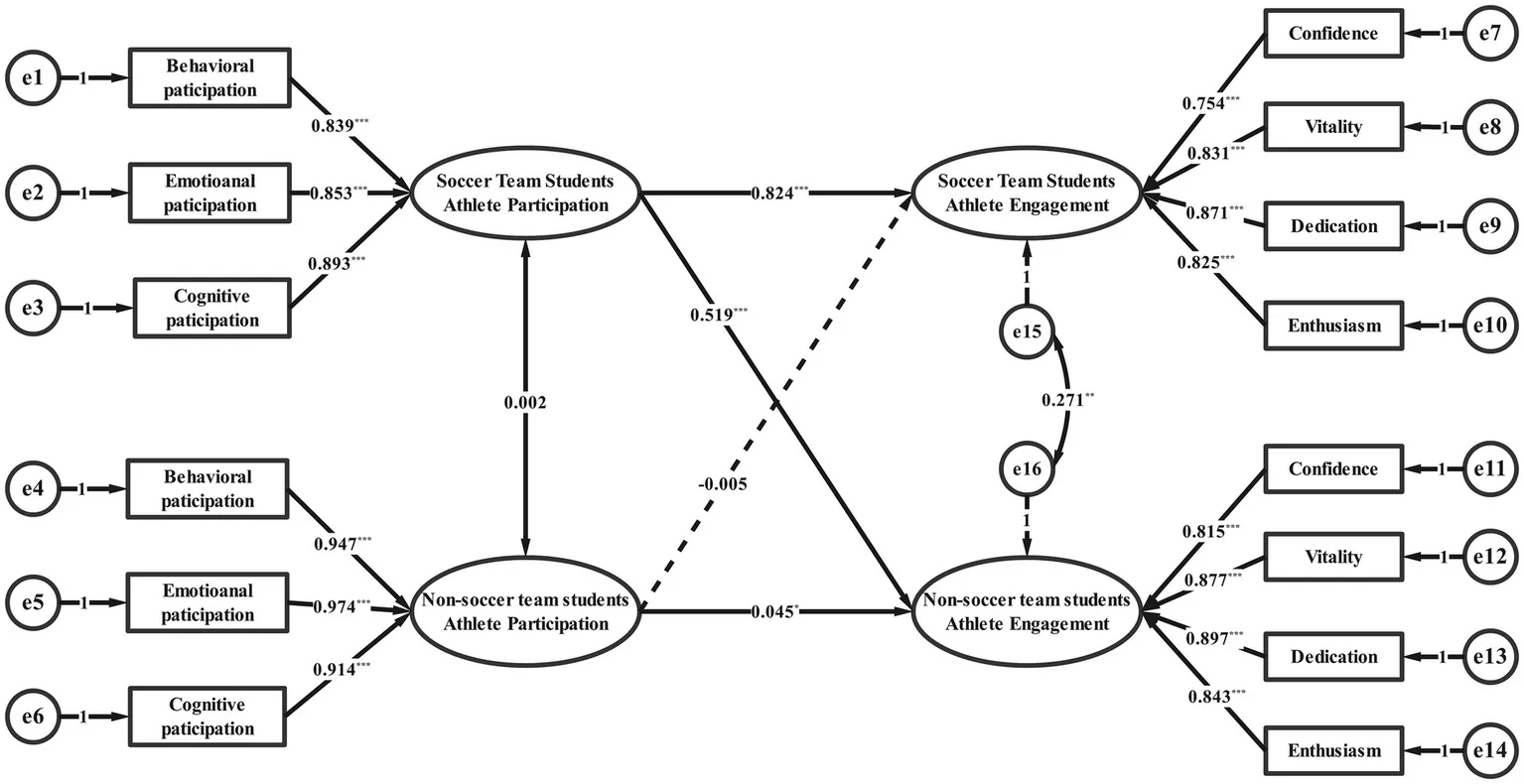
Results of the actor-partner interdependence model analysis. All coefficients are standardized. *p < 0.05; **p < 0.01; ***p < 0.001.

The Actor–Partner Interdependence Model (APIM) analysis is performed using Amos 26.0. The results show a good model fit: *χ*^2^ = 471.753, CFI = 0.985, TLI = 0.981, RMSEA = 0.051, SRMR = 0.020. Soccer team students and non-soccer team students can be distinguished based on whether they have been consistently and stably involved in soccer activities. This study first tests the standard APIM for distinguishable dyads. The APIM results are shown in Figure 2. The results indicate that the actor effect for soccer team students is significant (*β* = 0.824, *p* < 0.001). The actor effect for non-soccer team students is statistically significant but trivial in magnitude (*β* = 0.045, *p* < 0.05). In contrast, the partner effect of non-soccer team students is significant (*p* < 0.001). Moreover, the partner effect for non-soccer team students is substantially greater than their actor effect.

In the model, the actor and partner effects are constrained to be equal across soccer team students and non-soccer team students. The results show that the chi-square difference test is significant (Δχ^2^ = 3203.07, *p* < 0.001). The model does not support the hypothesis of equal actor and partner effects for soccer team students and non-soccer team students (39). Therefore, in further dyadic pattern analyses, soccer team students and non-soccer team students can still be treated as distinguishable dyads.

This study uses bootstrap resampling with 5,000 iterations to estimate the confidence interval for the k value. The results indicate that the k value for soccer team students is −0.003 [95% CI (−0.015, 0.010)]. Since 0 falls within the confidence interval, the dyadic pattern among soccer team students is classified as an actor-only pattern (39). For non-soccer team students, the absolute value of the actor effect is less than 0.1. Therefore, the k value is not calculated (42). Moreover, both the actor and partner effects of non-soccer team students are significant. The partner effect is substantially larger than the actor effect. Following Fitzpatrick et al. (43), this pattern is classified as a mixed pattern.

## 4 Discussion

Based on social interaction theory, this study uses the Actor-Partner interdependence model to investigate the influence of athlete participation on athlete engagement among different groups of adolescent students. The results show that soccer team students’ athlete participation significantly and positively predicts their own athlete engagement. Meanwhile, it also influences the athlete engagement of non-soccer team students. Non-soccer team students’ athlete participation positively predicts their own athlete engagement. But their athlete participation does not significantly influence soccer team students’ athlete engagement. This study supports the use of soccer team students as peer leaders. These students can encourage other adolescents at schools to remain engaged in sports. This approach may help address insufficient physical activity among Chinese youth.

### 4.1 Actor effect of soccer team students

The result of this study shows that the actor effect for soccer team students is significant. Thus, H1a is supported. This finding is consistent with previous studies (44). According to social interaction theory, when team members recognize the importance of cooperation in achieving shared goals, their engagement may increase (45). Soccer team students have some characteristics, such as a high level of cooperation and clear goals (46). When they participate in soccer, they communicate and cooperate with their teammates to achieve team goals (47). This encourages them to exert more effort during training and competition and promotes their athlete engagement (48). Through this process, soccer team students may also strengthen their connections with one another (49). This connection may lead them to view training and competitions as tasks that require sustained effort rather than as simple sports activities. In addition, to achieve team goals and complete tasks, soccer team students need to show more enthusiasm and invest more energy in their athlete participation (50). This may improve their athlete engagement. In contrast, if soccer team students show a low level of athlete participation, this may affect team outcomes. This may affect the achievement of shared team goals. Social interaction theory emphasizes cooperation and shared goals. Therefore, athlete participation may predict athlete engagement among soccer team students.

### 4.2 Partner effect of soccer team students

The result indicates that the partner effect for soccer team students is not significant. Thus, H1b is not supported. This finding is not consistent with previous studies (51). According to social interaction theory, the behavior of one group may influence another group; this effect depends on how closely two groups’ goals and outcomes are linked (52). Although soccer team students and non-soccer team students are situated within the same school environment, the two groups may differ in their goals for soccer participation (53, 54). Soccer team students receive systematic training over an extended period. They may participate in soccer to improve competitive ability and achieve better results in competitions (55). In contrast, especially in China, non-soccer team students participate in soccer mainly to meet the physical education course requirements (56). Based on this goal, athlete participation among non-soccer team students may have little influence on soccer team students’ behaviors (57). Although non-soccer team students also participate in soccer at the behavioral, emotional, and cognitive levels, their goals may differ from those of soccer team students. Their level of interdependence may be low (58, 59). The two groups may be only weakly connected because they have different goals. Therefore, non-soccer team students’ athlete participation is unlikely to predict soccer team students’ athlete engagement.

### 4.3 Actor effect of non-soccer team students

The result of this study shows that the actor effect for non-soccer team students is significant. Thus, H2a is supported. This finding is consistent with previous studies. Social interaction theory suggests that individuals are more likely to continue a behavior if it is consistently encouraged (15). Specifically, in the school sports context, non-soccer team students may gain some positive experiences through participating in soccer (60–62). When they participate in soccer, non-soccer team students have more opportunities to practice sport skills (63). This makes them experience their personal progress and gradually gain recognition from others (64). When they recognize the positive value of participating in soccer, their understanding of soccer may strengthen. This may increase their athlete engagement (65). This may be important for non-soccer team students. In contrast to soccer team students, non-soccer team students do not need to maintain participation in soccer for competitions (66). When they receive positive encouragement and recognize the value of participating in soccer, they may become more emotionally invested and exert greater effort in sports. Therefore, based on social interaction theory, non-soccer team students’ athlete participation may influence their own athlete engagement.

### 4.4 Partner effect of non-soccer team students

The result shows that the partner effect of ordinary school students is significant. Thus, H2b is supported. Social interaction theory emphasizes that a reference group plays an important role in social interaction (26). It is used for self-evaluation, behavioral comparison, and meaning construction (67–69). In school settings, a reference group plays three primary roles. It provides a reference standard, behavioral guidelines, and a source of meaning (70, 71). First, from the perspective of reference standards, soccer team students represent an outstanding group in the sports field (72). During soccer participation, soccer team students may serve as a reference standard for non-soccer team students (73). This reference standard may cover some aspects of soccer participation. It includes technical and tactical abilities, as well as attitudes toward athlete participation (74). Based on this reference standard for athlete participation, non-soccer team students may evaluate and adjust their attitudes toward soccer (75). This may increase their athlete engagement. Second, from the perspective of behavioral guidelines, soccer team students and non-soccer team students are classmates (76). Non-soccer team students may observe and imitate the behaviors of soccer team students (77). The athlete participation of soccer team students reflects their sustained effort and commitment to soccer (78). This may provide non-soccer team students with a clear and easy to understand behavioral standard. Finally, from the perspective of a source of meaning, athlete participation among soccer team students may demonstrate the social value of soccer participation and provide non-soccer team students with a model to follow (79). These factors may help non-soccer team students understand the value of soccer and become more emotionally invested in soccer participation. They may also devote more energy and attention to it (80). Thus, based on the three aspects of a reference group in social interaction theory, the athlete participation of soccer team students may predict the athlete engagement of non-soccer team students.

### 4.5 Implications and limitations

In practice, our findings provide practical guidance for the development of school physical education. Previous studies confirm that soccer team students may play a unique role in improving athlete engagement among adolescent students (81). Thus, given the insufficient athlete participation among adolescents, schools may promote campus physical activity by strengthening the development of school soccer teams (82). Meanwhile, teachers should develop targeted teaching and training strategies for different student groups. For soccer team students, coaches should emphasize their roles and responsibilities during daily training and help them understand the importance of team membership (83). For non-soccer team students, teachers should establish formal mentoring programs in regular physical education classes. In these programs, soccer team students can provide guidance and encouragement to non-soccer team students. This can strengthen mutual engagement among students and reinforce positive values related to sports.

This study has certain limitations that need to be acknowledged. First, the sample selection in this study may be subject to a certain degree of selection bias. This is because each non-soccer team student is paired with a soccer team student based on a naturally formed close friendship. These non-soccer team students receive long-term support in sports from soccer team students. They may not be fully representative of the broader general student population. These long-term friendships may interfere with the partner effect among non-soccer team students. Therefore, future research may include friendship duration and closeness as moderators in the model. This may make the findings more convincing. Meanwhile, future studies should select a broader and more diverse group of students as the sample. This can help determine whether soccer team students’ athlete participation influences the athlete engagement of other student groups. Second, the study sample comes from 167 designated campus soccer schools. However, existing analyses have mainly focused on actor and partner effects at the student-dyad level. Different schools may differ in resource allocation, soccer atmosphere, and culture. These factors may affect students’ athlete participation and athlete engagement. Therefore, the path relationships in the study model and their statistical significance may be influenced by these factors to some extent. Future studies should use multilevel actor-partner interdependence models or cluster-robust estimation methods. School factors should be included in the analysis to improve the reliability of the model estimates. Finally, this study adopts a cross-sectional actor-partner interdependence analysis design, which limits its ability to explicitly delineate the causal relationships among variables. Future studies should use a longitudinal actor–partner interdependence model. This approach can help researchers better understand the causal relationship between sports participation and sports engagement.

## 5 Conclusion

This study demonstrates that athlete participation influences athlete engagement across adolescent student groups. Soccer team students’ athlete engagement is mainly driven by their own participation experiences in structured soccer settings. In contrast, non-soccer team students’ athlete participation shows only a small actor effect. Their athlete engagement is more strongly shaped by the participation and role-modeling influence of soccer team students. These findings extend the understanding of adolescent athlete engagement from an individual-level process to a peer-influence process. In Chinese school sports, strengthening campus soccer teams may be an important strategy. Using soccer team students as peer leaders may also be helpful. These approaches may encourage other students to remain engaged in sports.

## Data Availability

The raw data supporting the conclusions of this article will be made available by the authors, without undue reservation.
